# Biohybrid robot with skeletal muscle tissue covered with a collagen structure for moving in air

**DOI:** 10.1063/1.5127204

**Published:** 2020-04-01

**Authors:** Yuya Morimoto, Hiroaki Onoe, Shoji Takeuchi

**Affiliations:** 1 Institute of Industrial Science (IIS), The University of Tokyo, 4-6-1 Komaba, Meguro-ku, Tokyo 153-8505, Japan; 2 Department of Mechano-Informatics, Graduate School of Information Science and Technology, The University of Tokyo, 7-3-1 Hongo, Bunkyo-ku, Tokyo 113-8656, Japan; 3 Department of Mechanical Engineering, Faculty of Science and Technology, Keio University, 3-14-1 Hiyoshi, Kouhoku-ku, Yokohama-shi, Kanagawa 223-8522, Japan; 4 International Research Center for Neurointelligence (WPI-IRCN), The University of Tokyo Institutes for Advanced Study (UTIAS), The University of Tokyo, 7-3-1 Hongo, Bunkyo-ku, Tokyo 113-0033, Japan

## Abstract

Biohybrid robots composed of biological and synthetic components have been introduced to reconstruct biological functions in mechanical systems and obtain better understanding of biological designs. For example, biohybrid robots powered by skeletal muscle tissue have already succeeded in performing various movements. However, it has been difficult for the conventional biohybrid robots to actuate in air, as the skeletal muscle tissue often dries out in air and is damaged. To overcome this limitation, we propose a biohybrid robot in which the skeletal muscle tissue is encapsulated in a collagen structure to maintain the required humidity conditions when operated in air. As the skeletal muscle tissue maintains high cell viability and contractility, even after encapsulation within the collagen structure, the biohybrid robot can move in air through contractions of the skeletal muscle tissue. To demonstrate the applicability of the developed biohybrid robot, we demonstrate its use in object manipulation. In addition, to prove its capability of functionality enhancement, we show that the biohybrid robot can actuate for a long term when perfusable tubes are set inside the collagen structure; it can actuate even while culturing cells on its surface. The developed biohybrid robot composed of skeletal muscle tissue and collagen structure can be employed within platforms used to replicate various motions of land animals.

## INTRODUCTION

Biohybrid robots constructed through the integration of biological components and synthetic structures have received a great deal of interest, as a method to incorporate biological functions into mechanical systems and study biological designs *in vitro.*^1–3^ Muscle tissue is one of the potential candidates used as the driving element of biohybrid robots owing to its high contraction efficiency and superior power-to-weight ratio of contractions.^2,4^ The principle driving mechanism of a biohybrid robot with muscle tissue established on a flexible substrate is the deformation of the substrate through muscle contractions.^5,6^ By inducing deformation of the substrate, under changing configurations and dimensions, biohybrid robots have succeeded in performing various biomimetic motions such as pumping,^7,8^ gripping,^9,10^ walking,^9,11,12^ and swimming.^13–16^ In addition, recent developments in the *in vitro* fabrication techniques for muscle tissue allow the construction of biohybrid robots with an antagonistic pair of skeletal muscle tissues, to enable bidirectional motions controlled by selective contractions of each muscle tissue.^17,18^ Although the muscle tissue allows the biohybrid robots to perform various motions, the available environments for the biohybrid robots are limited to culture media or cell-culture buffers, as muscle tissue can be activated only in a liquid environment.

As a solution to drive a biohybrid robot in air, encapsulation of the muscle tissue is deemed as a promising technique to enable isolation within an environment with the required humidity conditions. Akiyama *et al.* developed a biohybrid gripper with muscle tissue packaged in a polydimethylsiloxane (PDMS) case with its culture medium. This biohybrid gripper could drive in air without drying up the muscle tissue.^19^ Although the study successfully demonstrated the importance of encapsulating muscle tissue in a culture medium, for operating a biohybrid robot in air, the biohybrid gripper could not drive in an arbitrary position in air, as human bodies could, because the PDMS case was of a tabletop form with no sealing upper surface.

**FIG. 1.**
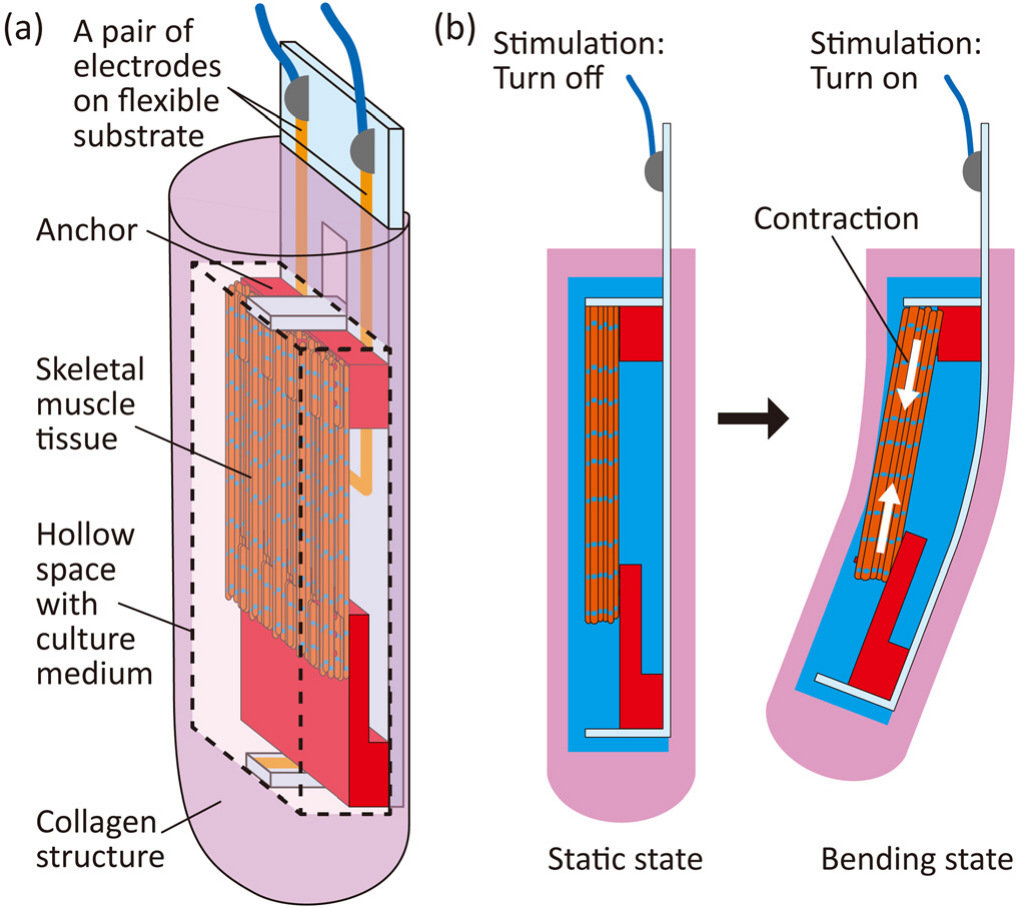
Concept of a biohybrid robot with a skeletal muscle tissue encapsulated with a collagen structure. (a) Illustration of a biohybrid robot with a skeletal muscle tissue encapsulated in a collagen structure. (b) Motion of the biohybrid robot enabled by contractions of the skeletal muscle tissue in the collagen structure.

In this study, we develop a biohybrid robot with skeletal muscle tissue encapsulated in a collagen structure, which can drive in any position in air (Fig. 1). In the case of a biohybrid robot driving in air, the skeletal muscle tissue and a pair of electrodes to stimulate the tissue are placed in the culture medium in a hollow space within the collagen structure. Therefore, the skeletal muscle tissue can be maintained under the required humidity conditions and can contract according to the applied electrical stimulations when the biohybrid robot is placed anywhere in air. Moreover, the hollow space facilitates smooth contractions of the skeletal muscle tissue, inducing deformation of the collagen structure, because there is no collagen touching the muscle tissue, which can interfere with the muscle contractions. In this work, we evaluate the motion properties of the biohybrid robot driving in air and demonstrate that the biohybrid robot can manipulate a bead through the deformation of the collagen structure triggered by contractions of the skeletal muscle tissue. Furthermore, as examples of other possible types of biohybrid robots implementing additional functions, we investigate the actuation properties when perfusable tubes are mounted on the robot and when a cell layer is formed on the robot.

## RESULTS AND DISCUSSION

### Construction of a biohybrid robot driving in air

**FIG. 2.**
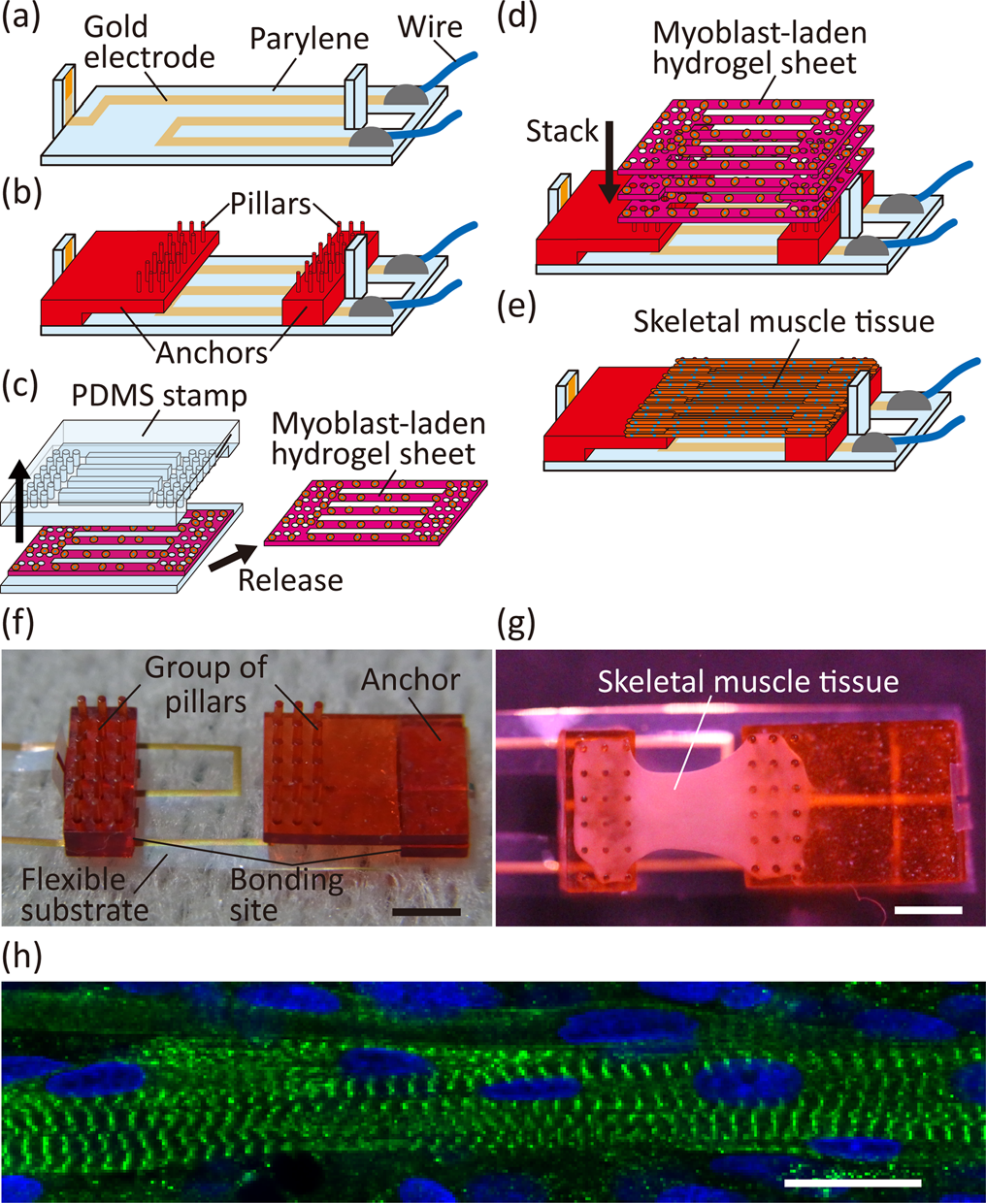
Construction of a skeletal muscle tissue on a flexible substrate. (a) Preparation of the flexible substrate through the parylene-based standard photolithography and bonding conductive wires with the conductive resin. (b) Bonding of anchors on the flexible substrate. (c) Formation of a myoblast-laden hydrogel sheet using a PDMS stamp. (d) Stack of myoblast-laden hydrogel sheets on the anchors on the flexible substrate. (e) Formation of the skeletal muscle tissue on the anchors. (f) Image of the flexible substrate with the anchors. (g) Image of the fabricated device with a skeletal muscle tissue on the substrate. (h) Confocal microscopic image of the skeletal muscle tissue. The α-actinin is indicated in green, and the cell nuclei are indicated in blue. Scale bars, (f) and (g) 2 mm and (h) 20 *μ*m.

The fabrication process of a biohybrid robot, with skeletal muscle tissue encapsulated in the collagen structure, involved three steps: (i) preparation of a flexible substrate with electrodes and anchors, (ii) formation of skeletal muscle tissue on the flexible substrate, and (iii) encapsulation of the skeletal muscle tissue and electrodes within a collagen structure. In step (i), a flexible substrate with gold electrodes was fabricated via parylene-based standard photolithography [Figs. 2(a) and 2(b)]. By partially covering the parylene on the surface of the electrodes, the electrodes were insulated over the entire surface except at the tips. Anchors for the skeletal muscle tissue, fabricated through stereolithography, were bonded on the flexible substrate. As the electrode tip was placed on the side of each anchor, it was possible to apply electrical stimulations between the anchors. In addition, we confirmed that the distance between each bonding site of the anchor and substrate could be altered without changing the distance between each group of pillars on the anchor, by using an L-shaped anchor, which shifted the position of the pillar group relative to the bonding site [Fig. 2(f)]. By using anchors with different shapes, we succeeded in setting the distance between each bonding site, which is the deformable substrate length through contractions of skeletal muscle tissue, to 4, 6, and 8 mm, while maintaining the distance between each group of pillars at 4 mm. In step (ii), we stacked and cultured myoblast-laden hydrogel sheets on the anchors to construct skeletal muscle tissue [Figs. 2(d) and 2(e)], using a method previously explained.^17^ The shapes of the myoblast-laden hydrogel sheets were determined by sandwiching uncured hydrogel solution with myoblasts, using a PDMS stamp and a silicone rubber sheet. The hydrogel sheets had striped structures to facilitate the alignment of myoblasts,^17,18^ because muscle cells in the striped structure tend to align along the stripe direction.^20,21^ The hydrogel sheets were immobilized on the anchors by fitting holes at both edges of the sheets and pillars on the anchors. After 10 days of culturing the stacked myoblast-laden hydrogel sheets on the anchors [Fig. 2(g)], skeletal muscle tissue was formed on the anchors, through the fusion of the myoblasts and skeletal muscle fibers, similar to that occurring in the conventional methods.^17,18^ To investigate the morphology of the skeletal muscle tissue, we performed immunostaining of the α-actinin arrangements in the tissue. The image of α-actinin immunostaining showed that the striped patterns of α-actinin were formed in the tissue [Fig. 2(h)], indicating formation of sarcomeres. This result confirmed that the skeletal muscle tissue had the basic morphological features representing contractible striated skeletal muscle tissues.

**FIG. 3.**
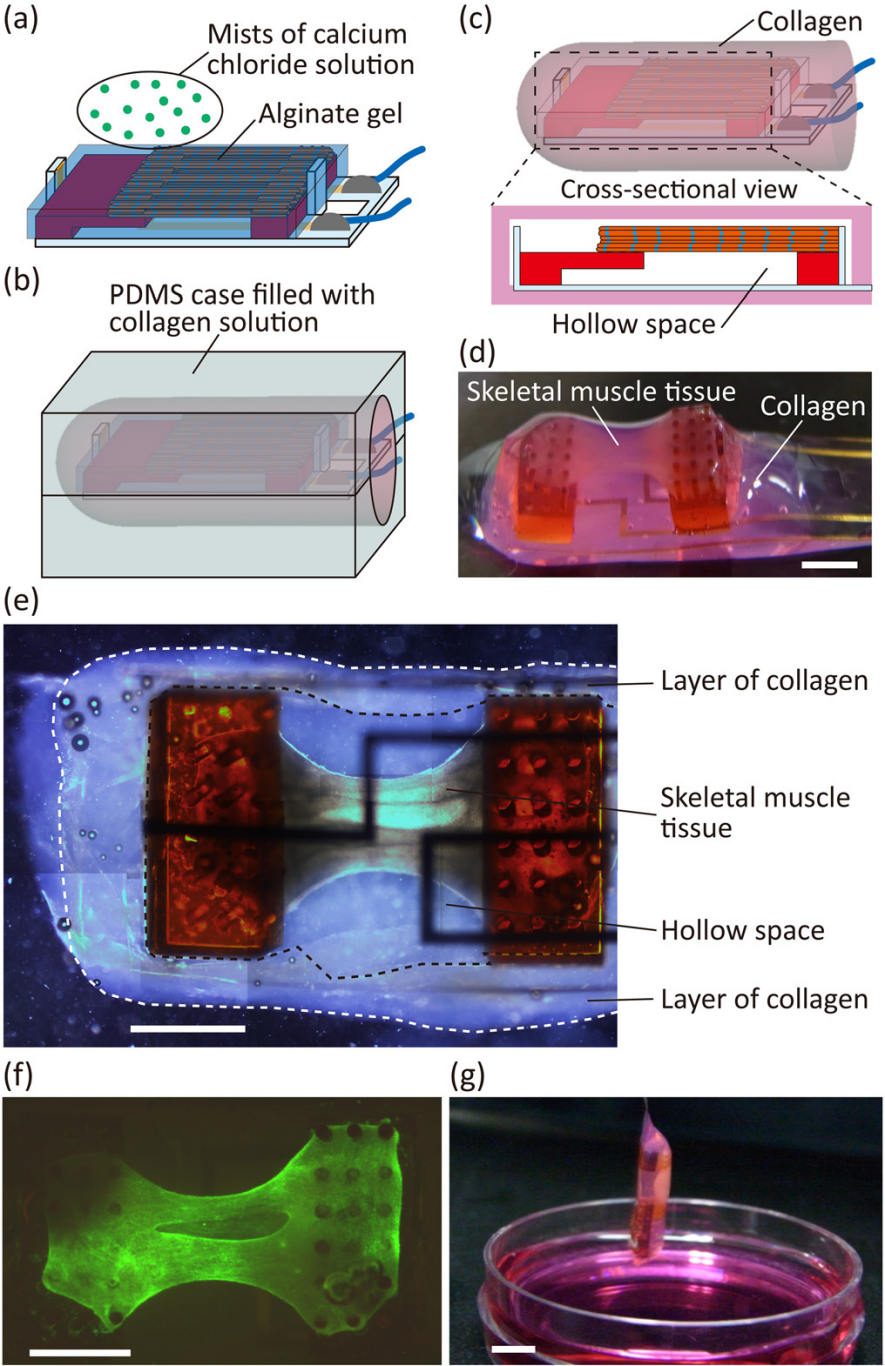
Construction of a biohybrid robot with the skeletal muscle tissue covered with the collagen structure. (a) Formation of the alginate gel around the skeletal muscle tissue and the pair of electrodes on the flexible substrate using mists of the calcium chloride solution. (b) Gelation of the collagen in a PDMS case to cover the alginate gel with the collagen. (c) Dissolving the alginate gel in the collagen structure to make the hollow space in it. (d) Image of the biohybrid robot with 4 mm deformable substrate length powered by the skeletal muscle tissue covered with the collagen structure. (e) Image of the inside of the collagen structure. (f) Fluorescent image of the skeletal muscle tissue stained with Live/Dead assay reagents in the biohybrid robot. The living cells are indicated in green, and the dead cells are indicated in red. (g) Image of the biohybrid robot with an 8 mm deformable substrate length in air. Scale bars, (d)–(f) 2 mm and (g) 5 mm.

In step (iii), we covered the skeletal muscle tissue and electrodes with alginate gel and embedded them into the hollow space of the collagen structure using the alginate gel as a sacrificial structure [Figs. 3(a)–3(c)]. The alginate gel was formed around the tissue and electrodes by inducing gelation of sodium alginate solution around them using a calcium chloride solution spray. By gelling the collagen covering the alginate gel in the PDMS mold and dissolving the alginate gel, we obtained the proposed biohybrid robot with skeletal muscle tissue and electrodes encapsulated in the collagen structure [Fig. 3(d)]. In this state, alginate polymers could diffuse out through the collagen structure because of the large pore size of collagen (one to several tens of micrometers in diameter).^22,23^ By analyzing the collagen structure, we confirmed that the skeletal muscle tissue and electrodes were in a hollow space, covered by a layer of collagen [Fig. 3(e)]. In addition, a cell-viability assay of the skeletal muscle tissue demonstrated that almost all cells were alive in the collagen structure [Fig. 3(f), supplementary material Fig. 1], indicating that the proposed method using alginate gel as a sacrificial structure was suitable for the encapsulation of skeletal muscle tissue within a collagen structure. As a result of the encapsulation, we were able to take out the biohybrid robot from the culture medium [Fig. 3(g)]. This result shows that the collagen structure did not break in air and that it kept covering the skeletal muscle tissue.

### Motion properties of the biohybrid robot

**FIG. 4.**
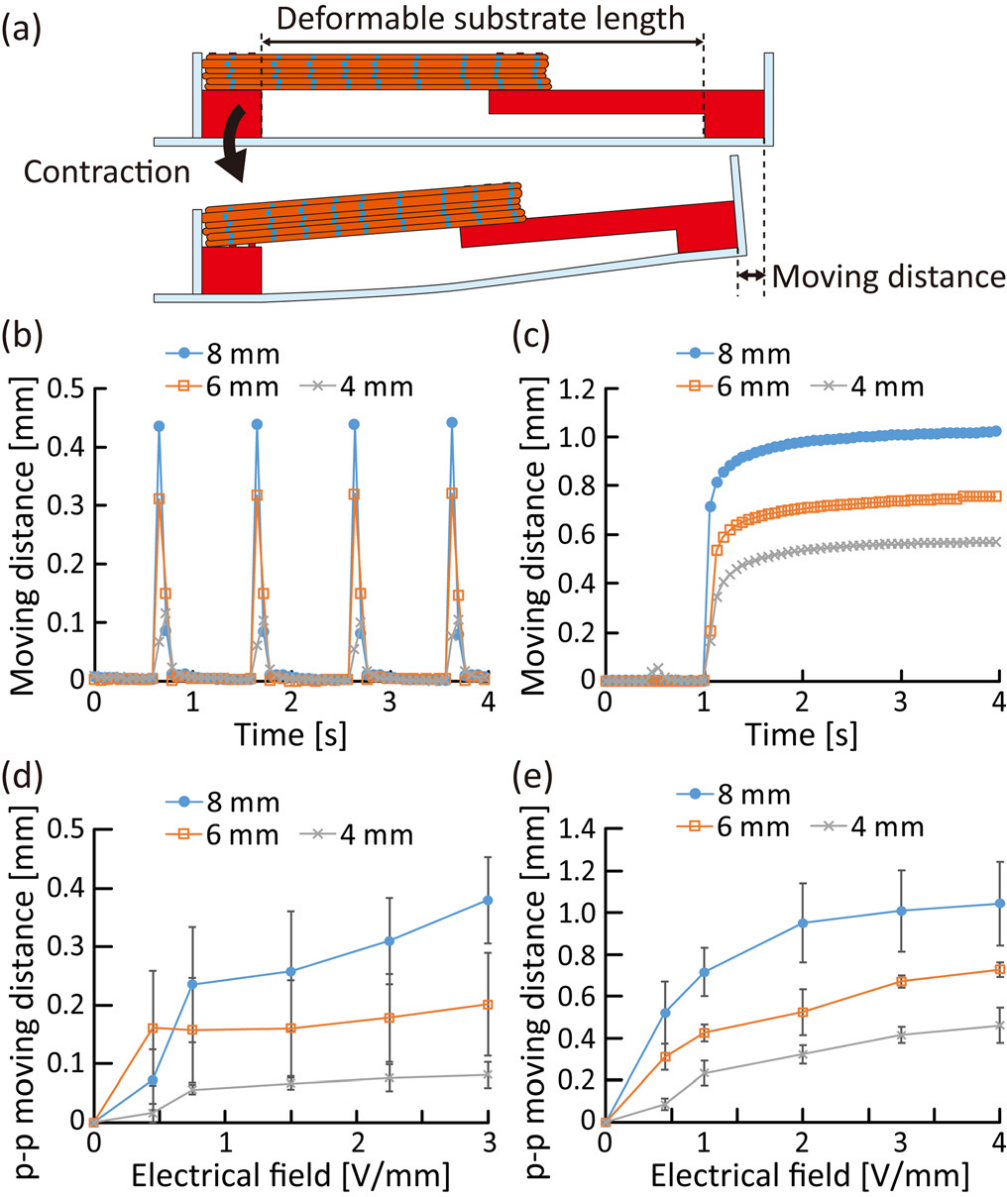
Evaluation of the deformation amount of the flexible substrate caused by contractions of the skeletal muscle tissue. (a) Conceptual illustration for measurements of the moving distance of the substrate tip caused by deformation of the substrate in the deformable substrate length. (b) and (c) Temporal variation of the moving distance according to different values of the deformable substrate length when applying electrical stimulations [electrical field, 3 V/mm; pulse duration, 2 ms; pulse frequency, (b) 1 Hz, (c) 50 Hz] to the skeletal muscle tissue. (d) and (e) The p–p moving distance of the substrate with different deformable substrate lengths (n = 3 devices), varied according to the magnitude of the electrical field applied [pulse duration, 2 ms; pulse frequency, (d) 1 Hz and (e) 50 Hz].

To evaluate the amount of deformation of the flexible substrate, caused by contractions of the skeletal muscle tissue, we investigated the moving distance of the anchor tip according to the deformable substrate length [Fig. 4(a)]; the deformable substrate length was controlled to 4, 6, and 8 mm, by using different shapes of anchors (supplementary material Fig. 2). To induce contractions, twitches, and tetanus of the skeletal muscle tissue, we applied electrical pulses (with a pulse duration of 2 ms) at 1 Hz and 50 Hz to the skeletal muscle tissue. When applying 1 Hz pulsed electrical stimulation, we confirmed that the deformations of the flexible substrate were synchronized with the 1 Hz twitches [Fig. 4(b)]. When applying electrical stimulations at 50 Hz, the deformations became larger because of the tetanus of the skeletal muscle tissue [Fig. 4(c)]. In addition, regardless of the twitches and tetanus, the moving distance of the tip increased as the deformable substrate length increased without changing that of the skeletal muscle tissue. These results show that the deformation amount of the flexible substrate can be altered by controlling the deformable substrate length, even when using skeletal muscle tissues with the same contractility. As tissue formation required a large number of cells, which was challenging to prepare,^24^ and as it was difficult to form a longer skeletal muscle tissue, we decided to change the deformation amount by controlling the deformable substrate length, instead of changing the muscle tissue length. Moreover, the peak-to-peak (p–p) moving distance induced by twitch and tetanus increased relative to the magnitude of the electrical stimulations, regardless of the deformable substrate length [Figs. 4(d) and 4(e)]. As the contractile force of the developed skeletal muscle tissue increased according to the magnitude of the electrical stimulations,^17,25^ the results indicate that the deformation amount of the flexible substrate was controlled by the muscle contractile force even when the deformable substrate length was different.

**FIG. 5.**
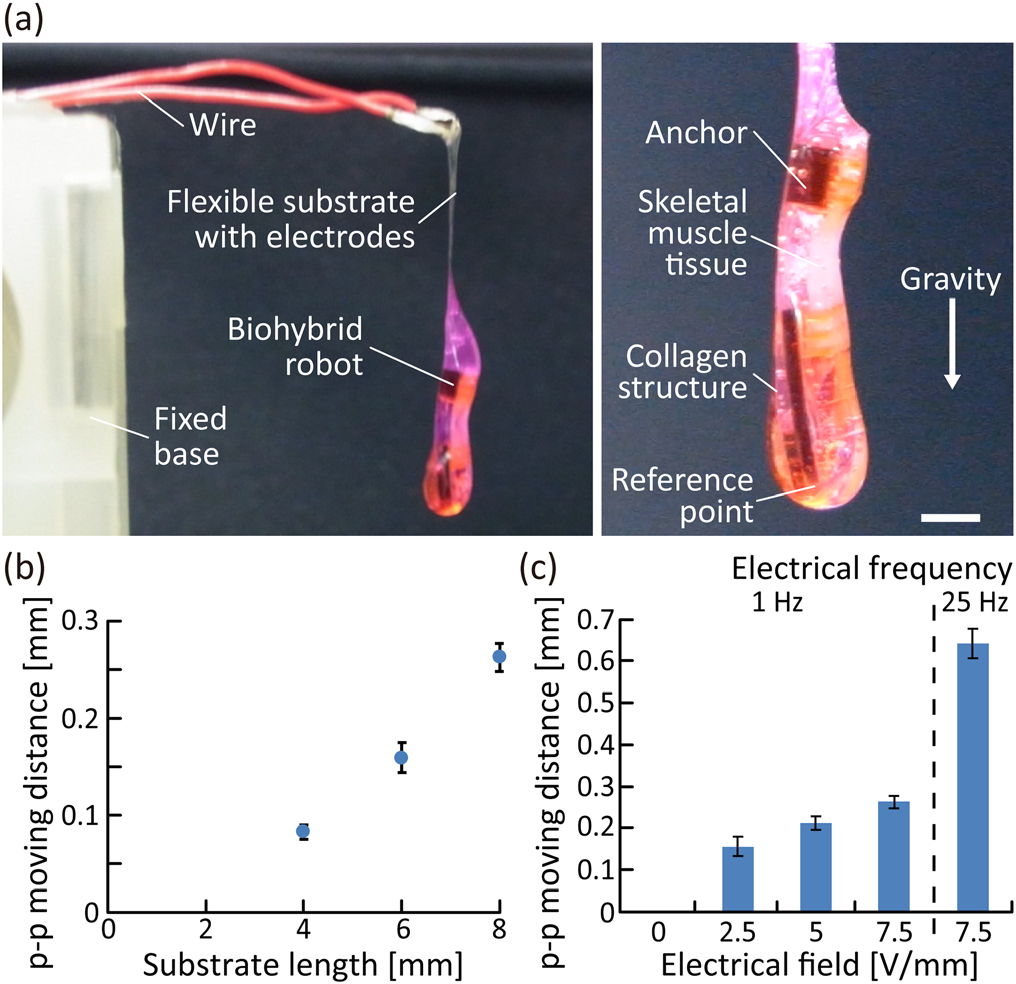
Actuations of the biohybrid robot in air powered by the skeletal muscle tissue. (a) Image of the biohybrid robot placed by immobilizing the wires onto the fixed base. (b) Variation of the p–p moving distance of the reference point of the biohybrid robot according to the deformable substrate length (n = 3 measurements) (electrical field, >6 V/mm; duration, 20 ms; frequency, 1 Hz). (c) Changes in the p–p moving distance caused by contractions of the skeletal muscle tissue induced by different electrical stimulations (n = 3 measurements) (duration, 20 ms). Scale bar, 2 mm.

Furthermore, we investigated the actuations of the biohybrid robots in air, driven by contractions of the skeletal muscle tissue. During the investigation, the biohybrid robot was placed in air by immobilizing conductive wires onto the fixed base [Fig. 5(a)]. As the skeletal muscle tissue was covered with a collagen structure to maintain the required humidity conditions even in air, the biohybrid robot was able to deform in accordance with the contractions of the skeletal muscle tissue when electrical stimulation was applied via a pair of electrodes in the collagen structure (supplementary material Movies 1 and 2). The result shows that the collagen structure worked effectively to maintain contractility of the skeletal muscle tissue in air because a skeletal muscle tissue that was not covered by the collagen structure lost its contractility when left in air (supplementary material Fig. 3). To analyze the actuations, we measured the moving distance of the reference point at the tip of the biohybrid robot. As a result, we confirmed that the moving distance of the biohybrid robot in air increased depending on the deformable substrate length, without any change in the properties of the skeletal muscle tissue [Fig. 5(b)]. Therefore, to increase the deformation amount, we decided to use a deformable substrate longer than the length of the skeletal muscle tissue, in the following experiments. Furthermore, using the biohybrid robot with a deformable substrate of length 8 mm, we evaluated the changes in the moving distance of the actuations in air, according to the properties of the electrical stimulations applied [Fig. 5(c)]; the p–p moving distance of the reference point increased as the magnitude of the electrical field increased or the frequency of electrical pulses increased. This result indicates that the actuations of the biohybrid robot in air could be altered by controlling the electrical stimulation properties. In addition, when comparing the p–p moving distances in the cases with and without using the collagen structure [Figs. 4(d), 4(e), and 5(c)], the p–p moving distance was found to be reduced by approximately 40% by the collagen structure. Based on the obtained results, we concluded that enough deformation could remain even though the collagen structure interfered with the actuations of the biohybrid robot.

### Demonstration of applicability of the biohybrid robot in air

**FIG. 6.**
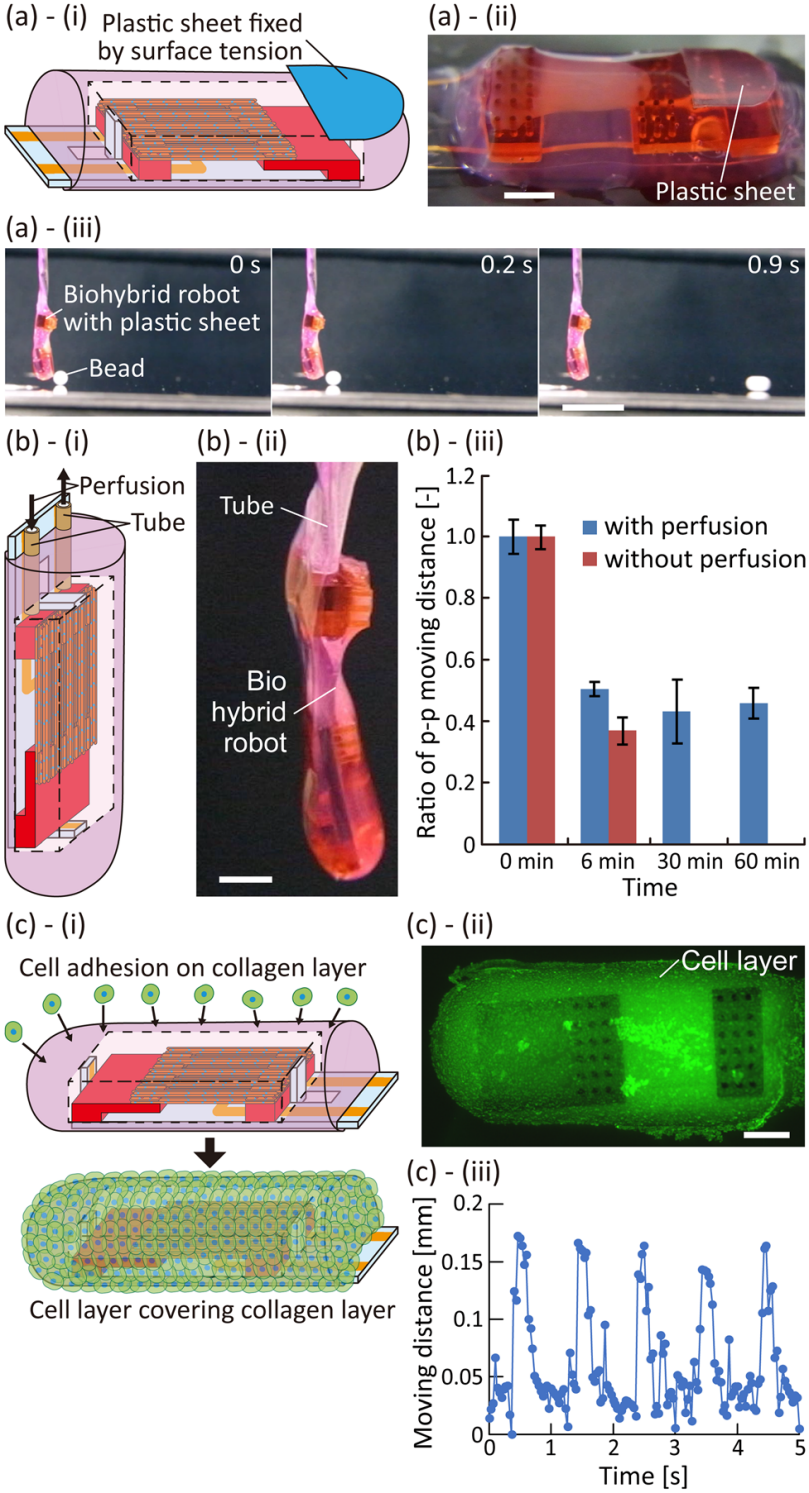
Demonstration of applicability of the biohybrid robot. (a) Pushing a bead in air using the biohybrid robot. (i) and (ii) Conceptual illustration and image of the biohybrid robot with a plastic sheet. (iii) Pushing a bead on the floor inclined approximately by 2° through actuations of the biohybrid robot with the plastic sheet when applying electrical stimulations for 1 s (electrical field, 2.5 V/mm; duration, 20 ms; frequency, 20 Hz). (b) Perfusion of the culture medium in the biohybrid robot. (i) and (ii) Conceptual illustration and image of the biohybrid robot with tubes used for perfusion. (iii) Variation in time of the p–p moving distance of the biohybrid robot with and without tubes (n = 3 measurements). (c) Biohybrid robot covered with NIH3T3 cells. (i) Conceptual illustration of the adhesion of NIH3T3 cells on the surface of the biohybrid robot. (ii) Image of the surface of the collagen structure covered with cells stained with reagents used to check the cell viability. The living cells are marked in green, and the dead cells are marked in red. (iii) Change in the moving distance when the skeletal muscle tissue was contracted by applying electrical stimulations (electrical field, 2.5 V/mm; frequency, 1 Hz; duration, 20 ms). Scale bars, (a)-(ii), (b)-(ii), (c)-(ii) 2 mm, (a)-(iii) 1 cm.

To demonstrate object manipulation in air using the biohybrid robot, we let the constructed biohybrid robot push a bead. In the demonstration, a plastic sheet was placed on the surface of the robot to serve as a contact surface to prevent the bead from adhering to the surface of the collagen structure because of the surface tension [Figs. 6(a)-(i) and 6(a)-(ii)]; the plastic sheet was immobilized on the surface owing to surface tension. After suspending the biohybrid robot with the plastic sheet, we placed a polystyrene bead of 2 mm diameter in front of the plastic sheet. In this state, electrical stimulations with high frequency were applied for 1 s to induce tetanus of the skeletal muscle tissue, so that the plastic sheet hit the bead because of the actuations of the biohybrid robot. When the bead was placed on a horizontal floor, although the bead was pushed by robot actuations, it did not roll because of the friction between the beads and the floor (supplementary material Movie 3). On the other hand, when the floor was inclined by approximately 2°, the bead rolled with the actuation of the biohybrid robot as a trigger [Fig. 6(a)-(iii), supplementary material Movie 4]. These results show that the biohybrid robot succeeded in pushing the bead using the contractions of the skeletal muscle tissues in air.

The biohybrid robot will gradually dry out in air, because the collagen structure will not be able to prevent evaporation of water, limiting the robot's long-term actuations. By placing tubes in the biohybrid robots to perfuse the culture medium, we were able to facilitate the long-term availability of the biohybrid robot in air [Figs. 6(b)-(i) and 6(b)-(ii)]. In the biohybrid robot with perfusable tubes, the tube ends were placed in the hollow space in the collagen structure; hence, the culture medium was stably perfusable without any collagen clogging the tube. Under the perfusion of the culture medium at 5 nl/s, we continuously applied 1 Hz pulsed electrical stimulations to induce twitches of the skeletal muscle tissue of the robot. As a result of the perfusion within the tubes, the biohybrid robot succeeded in performing continuous actuations for 1 h, in contrast to the case of the biohybrid robot without the perfusion, which stopped actuations after 15 min because of the damage to the skeletal muscle tissue caused by drying out [Fig. 6(b)-(iii)]. In addition, the moving distance decreased as time passed, even in the case of the biohybrid robot with perfusion of the culture medium. We assume that the decrease in the robot actuations was caused by the damage to the skeletal muscle tissue due to the electrolysis occurring at the electrodes. In addition, we consider that the spontaneous shrinkage of the skeletal muscle tissue caused by the intrinsic traction force also induced a decrease in the contractility.^11,17,26^ Therefore, the result indicates that perfusion of the culture medium allowed extension of the continuous actuation time of the biohybrid robot in air.

In addition, considering that cells could adhere onto the surface of the collagen structure, the biohybrid robot could be covered with a cell layer formed by cell culturing. To demonstrate the actuations of the cell-cultured biohybrid robot, we seeded NIH3T3 cells (mouse fibroblast-like cell line) on the surface of the collagen structure [Fig. 6(c)-(i)]. After 1 h of culturing, a layer of NIH3T3 cells with high viability was formed on the collagen structure [Fig. 6(c)-(ii)]. Although some parts of the cell layer were missing in this state [supplementary material Fig. 4(a)], we believe that the cell coverage could be improved by changing the culture conditions. To investigate the actuations of the biohybrid robot covered with the cell layer, we suspended it in air and applied 1 Hz pulsed electrical stimulations to induce muscle contractions. As a result, the biohybrid robot achieved actuations in air even with cells adhered to its surface [Fig. 6(c)-(iii)]. We compared the actuation performances of the biohybrid robots with and without the layer of NIH3T3 cells. The results of the comparison indicate that covering the biohybrid robot with the cell layer did not influence its actuation properties [supplementary material Fig. 4(b)]. Based on the obtained results, we believe that the biohybrid robot will actuate even when culturing epidermal keratinocytes on the surface of the collagen structure to form a cultured epidermal layer with water repellency. In addition, we confirmed that the deformation of the biohybrid robot occurred by the spontaneous shrinkage of the skeletal muscle tissue and coated NIH3T3 cells [supplementary material Fig. 4(c)], indicating that the cell layer did not improve the duration of robot actuation, as the spontaneous shrinkage induced a decrease in the muscle tissue contractility.

## CONCLUSION

In this study, we developed a biohybrid robot composed of skeletal muscle tissue and a flexible substrate with a pair of electrodes embedded in a hollow space of a collagen structure. As the collagen structure allowed maintaining the required humidity conditions around the skeletal muscle tissue even when the biohybrid robot was operated in air, applying electrical stimulations to the skeletal muscle tissue induced deformation of the biohybrid robot in air through its contractions. The deformation of the robot in air could be controlled according to the properties of the electrical stimulations applied, similar to that of a flexible substrate with skeletal muscle tissue in a culture medium. There is room to improve the actuation capabilities of the biohybrid robot. Although we used a flexible substrate with skeletal muscle tissue in the actuator configuration of the robot, the configuration would not be appropriate for large degrees of actuation, as conventional biohybrid robots using the configuration have shown that their moving distances are less than several hundreds of micrometers, similar to our biohybrid robot.^11,18^ In contrast, when mounting an antagonistic pair of skeletal muscle tissues on a skeleton with a joint, the biohybrid robot achieved ∼90° rotation of the joint, comparable to that of a living body.^17^ By arranging the actuator configuration with an antagonistic pair of skeletal muscle tissues into a collagen structure using the proposed method, the biohybrid robot with a collagen structure will allow large actuation in air.

Furthermore, we demonstrated that the biohybrid robot in air could push a bead by placing a plastic sheet on the surface of the robot and that it could actuate for a long term by embedding tubes into the hollow space of the collagen structure. From the applicability and long-term usability, we think that the biohybrid robot will be a useful model for actuations in air. In addition, we demonstrated that the biohybrid robot could actuate in air, even with fibroblasts covering the surface. We think that an epidermal layer with water repellency can be constructed on the robot by adhering epidermal keratinocytes instead of fibroblasts and culturing the keratinocytes at the air–liquid interface. Although the robot with a cell layer could not be driven for a long time owing to the spontaneous shrinkage under the configuration of the robot, previous research studies have shown that the spontaneous shrinkage can be prevented by using an antagonistic pair of skeletal muscle tissues.^17,18^ By combining the antagonistic pair of muscle tissues and the construction of the epidermal layer with our biohybrid robot, we believe that the techniques for our robot will enable the development of biohybrid robots driven in air for long-term with maintenance of humidity.

## METHODS

All rats used in this study were maintained in accordance with the policies of the University of Tokyo Institutional Animal Care and Use Committee (approval number: 30–2, IIS, the University of Tokyo).

### Preparation of the biohybrid robot with skeletal muscle tissue covered with a collagen structure

We first prepared a flexible substrate with anchors, using the modified method described previously.^18^ To do this, we first prepared a parylene sheet of 10 *μ*m thickness using a chemical vapor deposition machine (Parylene Deposition System 2010; Specialty Coating Systems Inc.) and then, formed gold electrodes on the sheet using standard photolithography. After connecting the gold electrodes to wires using conductive resin (Dotite D-723S; Fujikura Kasei Co., Ltd.), we formed a 2 *μ*m thick parylene layer on the parylene sheet excluding the tips of the gold electrodes, to insulate the photolithography pattern [Fig. 2(a)]. Subsequently, we prepared anchors coated with 2 *μ*m of parylene, using a 3D printer (Perfactory; EnvisionTEC) and the chemical vapor deposition machine. The anchors were bonded to the parylene sheet using a photoreactive medical adhesive (Loctite 3302; Henkel AG&Co. KGaA) [Fig. 2(b)]. During the process, using an L-shaped anchor (as either anchor), we controlled the deformable substrate length, in terms of the distance between each bonding site of the anchor and the substrate, without making any changes to the skeletal muscle tissue under development. For sterilization, after placing the sheet with anchors on a culture dish, we cleaned it using ethanol and exposed it to UV light for over 30 min in a sterilizing machine (Sterilizer FV-209B; As One Corporation). To facilitate cell adhesion, the anchors were coated with fibronectin (stabilized bovine fibronectin; Thermo Fisher Scientific Inc.).

To form the skeletal muscle tissue on the anchors, we used myoblast-laden hydrogel sheets fabricated with PDMS stamps; the fabrication method was described previously.^17^ Briefly, first, we prepared PDMS with grooves, through the solidification of 10:1 (base/elastomer) mixed PDMS elastomer (Sylgard 184 Silicone Elastomer, Dow Corning Toray Co., Ltd.) in a resin mold formed using the stereolithography machine. After treating the surface of the PDMS stamp with 2-methacryloyloxyethyl phosphorylcholine (MPC) polymers (NOF Corporation) to prevent cell adhesion, we sandwiched Matrigel solution (Corning Incorporated) containing 1 × 10^8^ cells/ml myoblasts between the PDMS stamp and a silicone rubber sheet (As One Corporation). The myoblasts were obtained as single-cell suspensions through mechanical treatments with type-II collagenase (Invitrogen) after preparing hind limbs of 1–2 days old Wistar neonatal rats (Sankyo Labo Service Corporation, Inc.). After the gelation of the myoblast-laden Matrigel in a 37 °C incubator and culturing for two days in the growth medium, which was Dulbecco's modified Eagle's medium (DMEM, Sigma-Aldrich Co., LLC.) with 10% fetal bovine serum (FBS, Japan Bioserum Co., Ltd.) and 1% penicillin–streptomycin (Invitrogen), we obtained the myoblast-laden hydrogel sheets with striped structures by releasing the PDMS stamp [Fig. 2(c)]. Subsequently, we stacked three myoblast-laden hydrogel sheets on the anchors [Fig. 2(d)] and induced the fusion of the myoblasts into skeletal muscle fibers by exchanging one half of the medium with the differentiation medium, DMEM with 2% horse serum (Sigma-Aldrich Co., LLC.) and 1% penicillin–streptomycin; this procedure was repeated daily. After 10 days of culturing the stacked sheets, skeletal muscle tissue was formed on the anchors.

To place the skeletal muscle tissue into the hollow space of the collagen structure, after releasing the flexible substrate with the skeletal muscle tissue from a culture dish, we encapsulated the tissue into a 1.5% sodium alginate solution (Wako Pure Chemical Industries, Ltd.) and gelled it with a mist of 150 mM calcium chloride solution (Kanto Chemical Col. Inc.) using a sprayer [Fig. 3(a)]. Subsequently, we placed it into a PDMS case shaped using a 3D printed resin mold and poured the collagen solution (Cellmatrix Type I-A; Nitta Gelatin Inc.) into the case [Fig. 3(b)]. After forming the collagen structure through the gelation of collagen in the PDMS case, we dissolved the alginate gel as a sacrificial structure using a differentiation medium with 40 *μ*g/ml alginate lyase (A1603; Sigma-Aldrich Co., LLC.) to create a hollow space in the collagen structure. As a result, the biohybrid robot with skeletal muscle tissue covered with a collagen structure was constructed [Fig. 3(c)].

### Cell staining

To evaluate the cell viabilities of the skeletal muscle tissue in the biohybrid robot, we stained it with 0.1% calcein AM and 0.2% ethidium homodimer (Live/Dead cell viability assay kit L3224; Invitrogen). To visualize α-actinin and the cell nuclei of the skeletal muscle tissue, the skeletal muscle tissue formed on the flexible substrate was washed using phosphate-buffered saline without Mg^2+^ and Ca^2+^ [PBS(−), Cell Science & Technology Institute, Inc.], fixed with 4% paraformaldehyde (Muto Pure Chemicals Co., Ltd.), permeabilized using 0.1% Triton X-100 (Alfa Aesar) in PBS(−) for 20 min, and then blocked with 2.5% bovine serum albumin (BSA, Sigma-Aldrich Co., LLC.) in PBS(−) overnight. Afterwards, we incubated the skeletal muscle tissue on the flexible substrate with PBS(−) containing 2.5% BSA and 0.1% monoclonal anti-α-actinin antibody (Sigma-Aldrich) at 4 °C, overnight. The skeletal muscle tissue was rinsed with PBS(−) and incubated using 0.1% Alexa Fluor conjugated secondary antibody (Thermo Fisher Scientific, Inc.) for 2 h at room temperature. After rinsing it with PBS(−) again, we stained the cell nuclei with 0.1% Hoechst 33342 (Invitrogen).

### Observations

To observe the flexible substrate with anchors and the skeletal muscle tissue on the substrate, we used a digital camera with a macro lens (EOS Kiss X6i; Canon Inc.,) to obtain a bright-field image and a confocal microscope (LSM780; Carl Zeiss). For observing the biohybrid robot, we used a macroscopic scope (MVX10; Olympus Corp.) to obtain fluorescent images of the robot and bright-field images of the collagen structure containing the skeletal muscle tissue. To obtain bright-field images and fluorescent images of the skeletal muscle tissue, we used a microscope (IX71N, Olympus Corp.).

### Motion analyses

To investigate contractility of the skeletal muscle tissue formed on the flexible substrate, we applied alternative-current (AC) square waves as electrical stimulations, generated using a function generator (Agilent Technologies Inc.) and an amplifier (Mess-Tek Co., Ltd.), to the skeletal muscle tissue via gold electrodes placed in the culture medium. To measure the moving distance of the tip of the flexible substrate, we observed the tip using a microscope (IX71N; Olympus) and quantified the movements of the tip using a motion analyzer (VW-H2MA; Keyence Corp.).

To evaluate the motions of the biohybrid robot in air, we placed it in air by immobilizing wires on a fixed base. In this state, we applied AC square waves to the skeletal muscle tissue via gold electrodes patterned on the flexible substrate. To estimate the moving distance of the reference point, we observed the point using the digital camera with a macro lens and quantified the movements of the tip using the motion analyzer.

### Object manipulation using the biohybrid robot

To demonstrate the ability of the biohybrid robot to perform object manipulation, a plastic sheet was prepared by cutting an overhead projector (OHP) film according to the shape of the robot. After placing the plastic sheet on the surface of the collagen structure [Fig. 6(a)-(i)], the biohybrid robot was set in air by immobilizing wires on the fixed base. After that, floors with different inclines, 0° and approximately 2°, were prepared and a polystyrene bead of 2 mm diameter was placed on the floor. By arranging the biohybrid robot and the bead, we let the biohybrid robot avoid touching the bead in its initial state, while allowing it to touch the bead during its actuation. The biohybrid robot was actuated by applying electrical stimulations using the same instruments used in the motion analyses.

### Actuations of the biohybrid robot with perfusable tubes

To enable perfusion of the culture medium in the hollow space within the collagen structure, we fabricated a biohybrid robot with perfusable tubes. In the fabrication, we first bonded silicone tubes on the flexible substrate using the photoreactive medical adhesive. After the formation of the skeletal muscle tissue on the flexible substrate with the tubes, we placed the ends of the tubes in the hollow space of the collagen structure by embedding them in the alginate gel, forming the collagen structure, and dissolving the alginate gel in the same manner as that in the fabrication of the biohybrid robot without perfusable tubes. To perfuse the differentiation medium at 5 nl/s in the hollow space, we infused and withdrew the medium using syringe pumps (KDS-210; KD Scientific Inc.) via the tubes. During perfusion, we applied electrical stimulations continuously via gold electrodes on the flexible substrate. We recorded its movements using the digital camera with the macro lens and measured the moving distance of its reference point using the motion analyzer.

### Actuations of the biohybrid robot covered with a cell layer

To demonstrate the actuations of the biohybrid robot covered with a cell layer, we first prepared suspensions of NIH3T3 cells (RIKEN Cell Bank) maintained in DMEM with 10% FBS (Biosera) and 1% penicillin–streptomycin in a 37 °C incubator. Subsequently, we adhered NIH3T3 cells on the surface of the biohybrid robot in the growth medium for myoblasts. During 1 h of culturing the NIH3T3 cells, we turned over the robot several times to facilitate adhesion of the cells onto the whole surface. After culturing, we transferred the robot with the cell layer into the new growth medium. We recorded and measured the motions of the biohybrid robot with the cell layer using the same approach employed in other motion analyses.

## SUPPLEMENTARY MATERIAL

See the supplementary material for the complete data of images, figures, and movies of the skeletal muscle tissue and biohybrid robot.

## References

[1] A. W. Feinberg , “ Biological soft robotics,” Annu. Rev. Biomed. Eng. 17, 243–265 (2015). 10.1146/annurev-bioeng-071114-040632 26643022

[2] L. Ricotti , B. Trimmer , A. W. Feinberg , R. Raman , K. K. Parker , R. Bashir , M. Sitti , S. Martel , P. Dario , and A. Menciassi , “ Biohybrid actuators for robotics: A review of devices actuated by living cells,” Sci. Rob. 2(12), eaaq0495 (2017). 10.1126/scirobotics.aaq0495 33157905

[3] V. Chan , H. H. Asada , and R. Bashir , “ Utilization and control of bioactuators across multiple length scales,” Lab Chip 14(4), 653–670 (2014). 10.1039/C3LC50989C 24345906

[4] L. Ricotti and A. Menciassi , “ Bio-hybrid muscle cell-based actuators,” Biomed. Microdevices 14(6), 987–998 (2012). 10.1007/s10544-012-9697-9 22960907

[5] J. U. Lind , T. A. Busbee , A. D. Valentine , F. S. Pasqualini , H. Y. Yuan , M. Yadid , S. J. Park , A. Kotikian , A. P. Nesmith , P. H. Campbell , J. J. Vlassak , J. A. Lewis , and K. K. Parker , “ Instrumented cardiac microphysiological devices via multimaterial three-dimensional printing,” Nat. Mater. 16(3), 303–309 (2017). 10.1038/nmat4782 27775708 PMC5321777

[6] Y. Sun , R. Duffy , A. Lee , and A. W. Feinberg , “ Optimizing the structure and contractility of engineered skeletal muscle thin films,” Acta Biomater. 9(8), 7885–7894 (2013). 10.1016/j.actbio.2013.04.036 23632372

[7] Y. Tanaka , K. Morishima , T. Shimizu , A. Kikuchi , M. Yamato , T. Okano , and T. Kitamori , “ An actuated pump on-chip powered by cultured cardiomyocytes,” Lab Chip 6(3), 362–368 (2006). 10.1039/b515149j 16511618

[8] J. Y. Park , I. C. Kim , J. G. Baek , M. S. Cha , J. S. Kim , S. H. Park , J. H. Lee , and B. K. Kim , “ Micro pumping with cardiomyocyte-polymer hybrid,” Lab Chip 7(10), 1367–1370 (2007). 10.1039/b703900j 17896023

[9] A. W. Feinberg , A. Feigel , S. S. Shevkoplyas , S. Sheehy , G. M. Whitesides , and K. K. Parker , “ Muscular thin films for building actuators and powering devices,” Science 317(5843), 1366–1370 (2007). 10.1126/science.1146885 17823347

[10] T. Hoshino and K. Morishima , “ Muscle-powered cantilever for microtweezers with an artificial micro skeleton and rat primary myotubes,” J. Biomech. Sci. Eng. 5(3), 245–251 (2010). 10.1299/jbse.5.245

[11] C. Cvetkovic , R. Raman , V. Chan , B. J. Williams , M. Tolish , P. Bajaj , M. S. Sakar , H. H. Asada , M. T. A. Saif , and R. Bashir , “ Three-dimensionally printed biological machines powered by skeletal muscle,” Proc. Natl. Acad. Sci. U. S. A. 111(28), 10125–10130 (2014). 10.1073/pnas.1401577111 24982152 PMC4104884

[12] R. Raman , C. Cvetkovic , S. G. M. Uzel , R. J. Platt , P. Sengupta , R. D. Kamm , and R. Bashir , “ Optogenetic skeletal muscle-powered adaptive biological machines,” Proc. Natl. Acad. Sci. U. S. A. 113(13), 3497–3502 (2016). 10.1073/pnas.1516139113 26976577 PMC4822586

[13] B. J. Williams , S. V. Anand , J. Rajagopalan , and M. T. A. Saif , “ A self-propelled biohybrid swimmer at low Reynolds number,” Nat. Commun. 5, 3081 (2014). 10.1038/ncomms4081 24435099

[14] J. C. Nawroth , H. Lee , A. W. Feinberg , C. M. Ripplinger , M. L. McCain , A. Grosberg , J. O. Dabiri , and K. K. Parker , “ A tissue-engineered jellyfish with biomimetic propulsion,” Nat. Biotechnol. 30(8), 792–797 (2012). 10.1038/nbt.2269 22820316 PMC4026938

[15] M. T. Holley , N. Nagarajan , C. Danielson , P. Zorlutuna , and K. Park , “ Development and characterization of muscle-based actuators for self-stabilizing swimming biorobots,” Lab Chip 16(18), 3473–3484 (2016). 10.1039/C6LC00681G 27464463

[16] S. J. Park , M. Gazzola , K. S. Park , S. Park , V. D. Santo , E. L. Blevins , J. U. Lind , P. H. Campbell , S. Dauth , A. K. Capulli , F. S. Pasqualini , S. Ahn , A. Cho , H. Y. Yuan , B. M. Maoz , R. Vijaykumar , J. W. Choi , K. Deisseroth , G. V. Lauder , L. Mahadevan , and K. K. Parker , “ Phototactic guidance of a tissue-engineered soft-robotic ray,” Science 353(6295), 158–162 (2016). 10.1126/science.aaf4292 27387948 PMC5526330

[17] Y. Morimoto , H. Onoe , and S. Takeuchi , “ Biohybrid robot powered by an antagonistic pair of skeletal muscle tissues,” Sci. Rob. 3(18), eaat4440 (2018). 10.1126/scirobotics.aat4440 33141706

[18] Y. Morimoto , H. Onoe , and S. Takeuchi , “ Biohybrid device with antagonistic skeletal muscle tissue for measurement of contractile force,” Adv. Rob. 33(5), 208–218 (2019). 10.1080/01691864.2019.1567382

[19] Y. Akiyama , T. Sakuma , K. Funakoshi , T. Hoshino , K. Iwabuchi , and K. Morishima , “ Atmospheric-operable bioactuator powered by insect muscle packaged with medium,” Lab Chip 13(24), 4870–4880 (2013). 10.1039/c3lc50490e 24185263

[20] Y. Morimoto , S. Mori , F. Sakai , and S. Takeuchi , “ Human induced pluripotent stem cell-derived fiber-shaped cardiac tissue on a chip,” Lab Chip 16(12), 2295–2301 (2016). 10.1039/C6LC00422A 27217209

[21] Y. Morimoto , M. Kato-Negishi , H. Onoe , and S. Takeuchi , “ Three-dimensional neuron-muscle constructs with neuromuscular junctions,” Biomaterials 34(37), 9413–9419 (2013). 10.1016/j.biomaterials.2013.08.062 24041425

[22] P. Banerjee , D. Lenz , J. P. Robinson , J. L. Rickus , and A. K. Bhunia , “ A novel and simple cell-based detection system with a collagen-encapsulated B-lymphocyte cell line as a biosensor for rapid detection of pathogens and toxins,” Lab. Invest. 88(2), 196–206 (2008). 10.1038/labinvest.3700703 18059364

[23] Y. L. Yang , S. Motte , and L. J. Kaufman , “ Pore size variable type I collagen gels and their interaction with glioma cells,” Biomaterials 31(21), 5678–5688 (2010). 10.1016/j.biomaterials.2010.03.039 20430434

[24] L. Moroni , J. A. Burdick , C. Highley , S. J. Lee , Y. Morimoto , S. Takeuchi , and J. J. Yoo , “ Biofabrication strategies for 3D in vitro models and regenerative medicine,” Nat. Rev. Mater. 3(5), 21–37 (2018). 10.1038/s41578-018-0006-y 31223488 PMC6586020

[25] Y. Yamamoto , A. Ito , H. Fujita , E. Nagamori , Y. Kawabe , and M. Kamihira , “ Functional evaluation of artificial skeletal muscle tissue constructs fabricated by a magnetic force-based tissue engineering technique,” Tissue Eng., Part A 17(1-2), 107–114 (2011). 10.1089/ten.tea.2010.0312 20672996

[26] H. Vandenburgh , J. Shansky , F. Benesch-Lee , V. Barbata , J. Reid , L. Thorrez , R. Valentini , and G. Crawford , “ Drug-screening platform based on the contractility of tissue-engineered muscle,” Muscle Nerve 37(4), 438–447 (2008). 10.1002/mus.20931 18236465

